# Development of a cDNA array for chicken gene expression analysis

**DOI:** 10.1186/1471-2164-6-13

**Published:** 2005-02-04

**Authors:** Joan Burnside, Paul Neiman, Jianshan Tang, Ryan Basom, Richard Talbot, Mark Aronszajn, David Burt, Jeff Delrow

**Affiliations:** 1 Delaware Biotechnology Institute, University of Delaware, 15 Innovation Way, Room 229, Newark, DE 19711 USA; 2 Division of Basic Sciences, Fred Hutchinson Cancer Research Center, 1100 Fairview Ave. N. C2-023, P.O. Box 19024, Seattle, WA 98109-1024 USA; 3 Bioinformatics Systems and Databases Glaxo Smith Kline King of Prussia, PA; 4 Genomics Resource, DNA Array Laboratory, Fred Hutchinson Cancer Research Center, 1100 Fairview Ave. N. C2-023, P.O. Box 19024, Seattle, WA 98109-1024 USA; 5 Dept. of Genomics & Bioinformatics Roslin Institute (Edinburgh) Roslin, Midlothian EH25 9PS, UK; 6 Biocomputing Resource, Fred Hutchinson Cancer Research Center, 1100 Fairview Ave. N. B1-080, P.O. Box 19024, Seattle, WA 98109-1024 USA

## Abstract

**Background:**

The application of microarray technology to functional genomic analysis in the chicken has been limited by the lack of arrays containing large numbers of genes.

**Results:**

We have produced cDNA arrays using chicken EST collections generated by BBSRC, University of Delaware and the Fred Hutchinson Cancer Research Center. From a total of 363,838 chicken ESTs representing 24 different adult or embryonic tissues, a set of 11,447 non-redundant ESTs were selected and added to an existing collection of clones (4,162) from immune tissues and a chicken bursal cell line (DT40). Quality control analysis indicates there are 13,007 useable features on the array, including 160 control spots. The array provides broad coverage of mRNAs expressed in many tissues; in addition, clones with expression unique to various tissues can be detected.

**Conclusions:**

A chicken multi-tissue cDNA microarray with 13,007 features is now available to academic researchers from genomics@fhcrc.org. Sequence information for all features on the array is in GenBank, and clones can be readily obtained. Targeted users include researchers in comparative and developmental biology, immunology, vaccine and agricultural technology. These arrays will be an important resource for the entire research community using the chicken as a model.

## Background

The chicken is an important experimental model for evolutionary and developmental biologists, immunologists, cell biologists, geneticists, as well as being an important agricultural commodity. The recent release of a draft of the chicken genome sequence, as well as the development of a large (531,351) collection of expressed sequence tags (ESTs) has dramatically changed the landscape for biologists wishing to use genomic tools to study the chicken. DNA microarrays are well accepted as an essential part of functional genomics. Several small chicken cDNA arrays have been fabricated and used in studies focused on the chicken immune system [1-4]. To enhance the utilization of existing resources and further develop the chicken as a model organism, a consortium was formed to produce microarrays using clones from the Biotechnology and Biological Sciences Research Council (BBSRC), University of Delaware (UD) and Fred Hutchinson Cancer Research Center (FHCRC). The BBSRC chicken cDNA project generated a large (>300,000) collection of ESTs that represents a wide range of adult and embryonic tissues [5]. The UD Chick EST project has focused on tissues important in agricultural production, with a heavy emphasis on the immune system [6]. The FHCRC EST collection was generated from DT40 cells (a transformed bursal cell line) [1,2], along with clones from the bursal EST project [7,8] and the UD activated T cell library [9]. By combining resources and clones from these projects, we have established a collection that encompasses a variety of tissues, and generated microarrays with 13,007 usable features. This paper describes the array with respect to clone selection and quality control parameters.

## Results and discussion

### Selection of clones for the array

**Figure 1:**
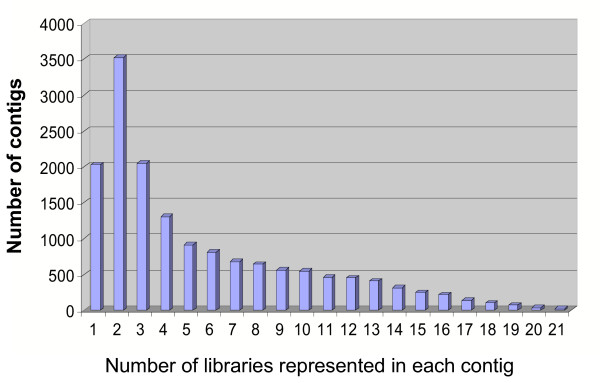
Library coverage in clones assembled into contigs. Clones from the BBSRC, UD, and FHCRC collections were assembled into 13,584 high scoring (BlastX>50) contigs using phrap software [10], and the number of different tissue libraries represented in each contig were scored. There were 6,832 contigs that had clones from 4 or more libraries, while the remaining contigs consisted of clones from 3 or fewer libraries.

A compilation of 363,838 chicken ESTs from the BBSRC, UD, and FHCRC collections were sorted into contigs (33,323) singlets (27,235), and singletons (8,794), using the default parameters of the phrap assembly program [10]. The phrap singletons contain sequences represented in the contig group, but could not be assembled, and were eliminated from further consideration. Both contigs and singlets groups were analyzed by using BlastX to compare to GenBank (nr) and BlastN to compare to human dbEST. Because of the evolutionary divergence between chicken and the majority of the sequences that populate GenBank, a Blast score >50 was considered a significant hit, and clones with scores<50 were excluded. Clones belonging to the existing chicken immunology collection (4,162 cDNAs from DT40 cells, bursa and lymphoid tissues) were sorted from the entire contig/singlet set, and after screening for *E.coli*, mitochondrial and ribosomal RNA contaminants, and identical Blast hits, a total of 2,248 and 13,584 singlets and contigs, respectively, remained as candidates from which to choose cDNAs for the final array. About half of the clones in the contig group were expressed in 4 or more libraries, indicating wide tissue expression (Figure 1). The remaining half was found in less than 3 libraries, indicating a more restrictive expression. For clones belonging to contigs, the most 5' clone was selected for inclusion on the array. This potentially introduces a 5' bias in the sequence available for hybridization; however, since the average insert size for all clones is approximately 1.2 kb and most cDNAs were made by oligo dT priming, clones should contain the entire downstream sequence.

**Figure 2:**
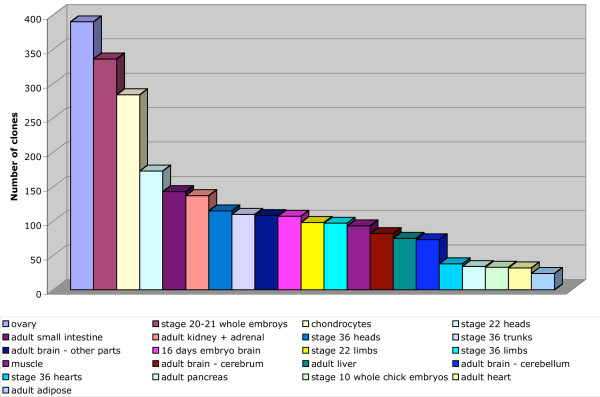
Library coverage of the singlets. Clones that are only represented once in the ESTs assembled with the phrap software [10], and with Blast scores>50, were analyze for distribution in the different libraries.

The library representation of the clones in the singlets group is shown in Figure 2. The numbers tended to reflect the depth of sequencing of the individual libraries [5,11]. The chondrocyte, ovary and stage 20–21 whole embryo libraries have more singlets; more than 25,000 ESTs were sequenced from each of these libraries, as opposed to 7–15,000 from the other libraries. The correlation is not perfect, however, and the lack of correspondence likely reflects similarities of some libraries to others in the collection, or relative specialization of the tissue, or a combination of these factors.

**Figure 3:**
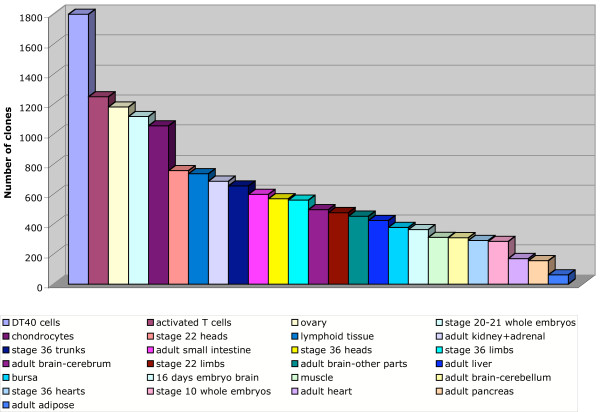
Library distribution in final clone set. The clones in the original immunology collection and clones randomly selected from the contigs and singlets were scored for library of origin.

**Figure 4:**
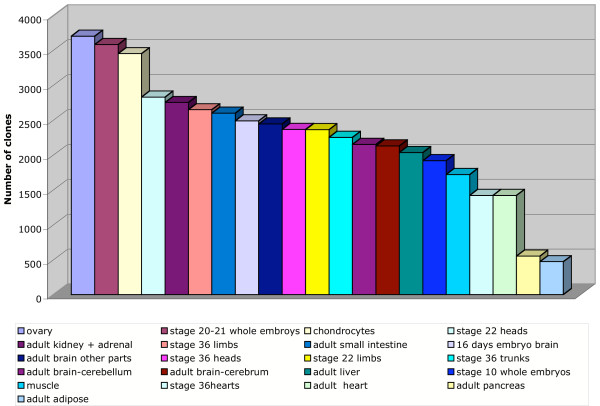
Minimal expected tissue coverage. The libraries represented in each of the contigs or singlets from which a clone was chosen for the array were scored to give an estimation of the expected coverage for a given tissue. Only clones in the BBSRC collection were included in this analysis.

The final selection of clones for the array was made by randomly choosing about 4,800 ESTs expressed in a wide range (>3) of tissues, and about 4,800 with a more narrow (1–3 tissues) expression profile, in addition to 1,735 singlets. The library distribution of the final clone selection is shown in Figure 3. However, it is important to note that because >50% of the clones were represented in multi-library contigs, the potential tissue representation on the array is greater than that depicted by library representation. Figure 4 shows the minimal expected tissue coverage of the 11,447 clones chosen from the BBSRC collection. Note, for example, that while only 724 clones from the stage 36 trunk library were selected for the array, at least 2,000 mRNAs from that tissue are represented by clones from various libraries.

### Annotation

A list of the clones can be accessed on-line [12]. The clones represented in the list total 15,769. PCR product quality was assessed using gel electrophoresis and the results were meticulously scored and recorded. After identifying poor quality PCR products (e.g., no detectable product, detection of multiple products), the number of useable features totals approximately 13,000, including control features. The annotation file contains accession numbers, source clone name, and source assigned annotation or Blast derived annotation. In addition the EST identification assigned by The Institute for Genome Research (TIGR) and found in TIGR's Gallus gallus Gene Index (GgGI) [13] is provided, as is the identifier for TIGR's consensus (TC) sequence and TIGR annotation. An analysis of the TC identifiers for clones on the array revealed that 1,184 mRNAs are represented by more than one clone. This is due to clones in non-overlapping contigs and some redundancy in the original immune collection. A more detailed annotation file, as well as a database for array data is under development and will be accessible on line [6].

Clone selection and array fabrication predated the sequencing of the chicken genome. An analysis of the sequence of the clones on the array indicates that 10,168 of the 21,447 predicted or annotated chicken genes in the GenBank chicken Unigene collection are present on the array. The remaining clones match cDNAs not yet included in Unigene, or other portions of the chicken genome, or are redundant.

Clones are available from their original source: the BBSRC collection, distributed by the MRC gene service [14]; the DKFZ collection at Heinrich-Pette-Institute maintained by Dr. Jean-Marie Buerstedde [15]; the DT40 collection at Fred Hutchinson Cancer Research Center, maintained by Dr. Paul Neiman [16]; the T-cell and lymphoid libraries, maintained by Dr. Joan Burnside of the Delaware Biotechnology Institute [6].

### Chicken 13K array performance

**Figure 5:**
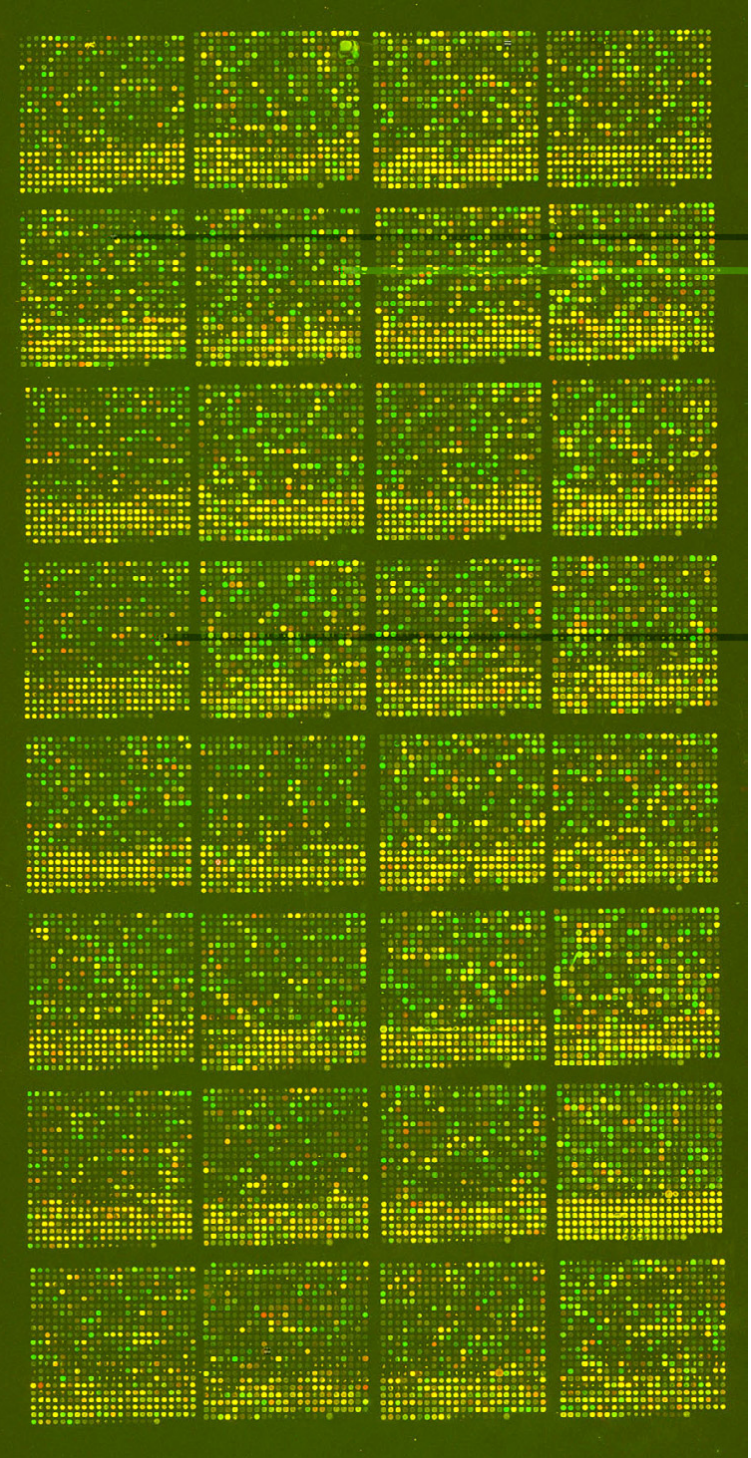
Image of 13K array hybridized to brain (Cy5™) and myc-transformed fibroblasts (Cy3™). The array layout is 32 blocks in a 4 × 8 configuration and each PCR product is represented once, with the exception of negative controls, which are replicated in each block.

An image of the 13K array hybridized to RNA extracted from chicken brain and myc-transformed embryo fibroblast samples and independently labeled with Cy3™ or Cy5™ fluorescent dyes is shown in Figure 5. There is good discrimination between the two samples, as well as many commonly expressed genes. Of noted prominence is the striking difference in signal intensities associated with the spots located near the bottom of each block. These spots correspond to clones represented in the DT40/UD/DKFZ immune collection, which were originally selected with a bias towards highly expressed genes. Since the BBSRC clones are predominantly from highly normalized libraries and were chosen as non-overlapping with the original immune system set, this resulted in a survey of lower abundance and more tissue-specific transcripts.

### Reproducibility

**Figure 6:**
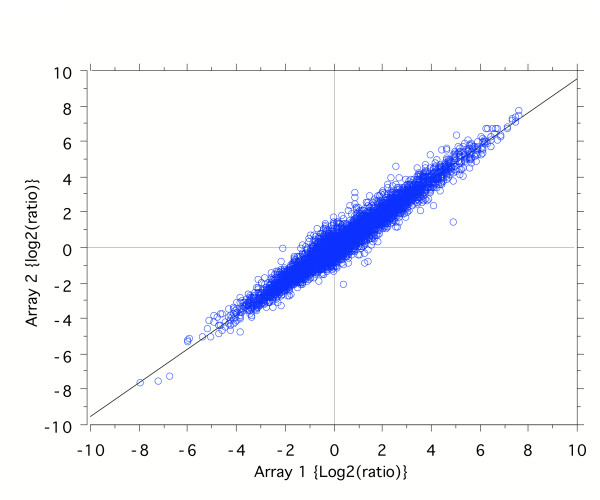
Correlation of signals from chicken 13k array hybridized to brain and fibroblast RNA. Samples were each labeled with Cy3™ and Cy5™ to perform a dye swap comparison.

Labeled samples were co-hybridized to the array for 16 hrs using standard protocols [12]. The same brain and fibroblast RNA extracts were also labeled by reversing the dye orientation and then co-hybridized to a second array. After image analysis, modest signal-to-noise (S/N) filtering, log base-2 ratio transformation, loess normalization, and corrections for the inverted dye orientations, the results from the two hybridizations were compared and were shown to be highly correlated (Figure 6; Pearson correlation coefficient, r = 0.972). The high correlation is indicative of a very high-level of technical reproducibility in array performance. Rare outlying data points and the slight deviation from a slope = 1, may reflect the influence of the different dyes used in the amino-allyl labeling.

### Signal-to-noise, specificity and sensitivity

We randomly chose one of the "myc-transformed embryo fibroblast vs. brain" array comparisons and determined the signal-to-noise (S/N) values for each channel using the background-corrected feature signals and the variation in the local background signal. Table 1 contains the results for the individual channels/samples. Of note is the high number of features with a S/N > 3.0, a value commonly used for defining the lower-bound threshold of detection. The mean S/N is also provided in Table 1 for each channel. These results reflect the significant detection capabilities obtainable in using the array. For example, the data from this representative comparison spanned the maximum fluorescent dynamic range of detection, from over 65,000 counts down to background count levels. In addition, the average local background signal for both channels was consistently low across the entire array, with no appreciable spatial block-level differences (see Table I). Furthermore, the variation in the local background signal was less than 38%. Taken collectively, the array provides a significant level of sensitivity for expression profiling.

**Table I:** Chicken 13K cDNA Microarray Performance Metrics

**Label / Sample**	**Mean BG Signal**	**Spot-Level S/N >3**	**Mean Spot-Level S/N**
Cy3 / Fibroblast	118 ± 6	88.0%	35.1
Cy5 / Brain	48 ± 3	86.3%	38.3

± standard deviation of the mean

**Figure 7:**
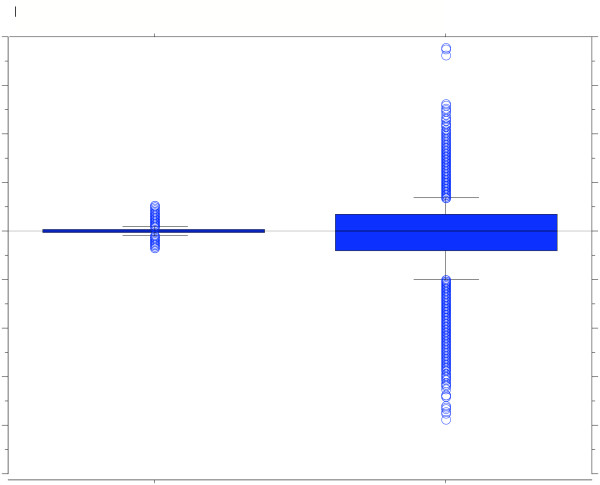
Box plot of Iog_2 _ratios from arrays hybridized to brain labeled independently with Cy5™ and Cy3™ and brain versus fibroblasts. The y-axis is the Iog_2_-transformed values for each comparison. The bar inside the box is the median value, the box upper and lower dimensions define the inter-quartile range, and the crossbars demark the 10th to 90th percentile range.

Figure 7 is a box plot of a "brain vs. brain" and a "myc-transformed embryo fibroblast vs. brain" comparison using the array. The y-axis is the Iog_2_-transformed (Cy3™/Cy5™) values for each comparison. The bar inside the box is the median value, the upper and lower dimensions of the box define the inter-quartile range, and the crossbars demark the 10th to 90th percentile range. The difference in the Iog_2 _ratio distributions between comparisons highlights the capabilities of the array to detect transcript-level differences between the fibroblast and brain samples.

**Figure 8:**
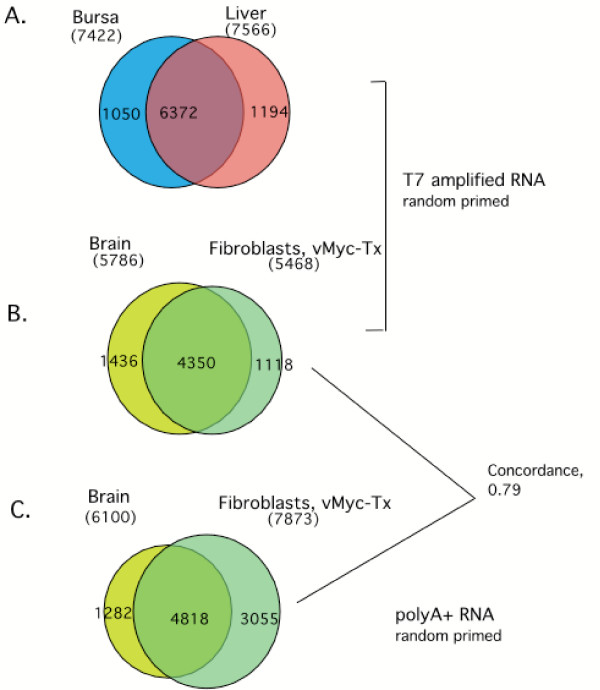
Performance of amplified RNA (aRNA) and mRNA from different tissues. Common and tissue specific expression is illustrated in panels A and B. Panel C shows fair concordance of hybridizing spots between aRNA and mRNA;

The Venn diagram in Figure 8 (A,B) indicates the sample-specific "detectable signals" (spot-level S/N >3.0) from bursa, liver, brain, and myc-transformed embryo fibroblast. Note that signals were obtained for 7,422 spots with RNA from bursa, suggesting that this array provides wide coverage for experiments with lymphoid tissues. Excellent coverage of liver, brain and fibroblast transcripts was obtained as well. The identification of tissue-specific transcripts is noteworthy and reflects the clone selection process, which was designed to provide detection of mRNAs in a wide range of tissues, as well as low abundance, unique transcripts. It is of interest that the myc-transformed fibroblasts are a quail derived cell line; these results indicate that these arrays will be useful for studies in other gallinaceous birds.

In a separate experiment, T7 amplified, random-primer labeled RNA was compared with random-primer labeled poly A RNA (from the same preparation). Figure 8C shows a fair concordance with about 80% of the same spots showing hybridization with each sample. However, this comparison reveals that amplification loses some signals detected with mRNA but picks up others, presumably from low abundance messages which amplify better (with respect to the cDNA sequences on the chip) than average. In another experiment (not shown) repeat amplifications of the same RNA prep give satisfactorily consistent results (correlation coefficient >0.9). These results emphasize that it is important to use the same method of RNA preparation and labeling to obtain reliable comparisons.

## Conclusions

An international consortium of researchers interested in using the chicken as both a model biological system and as an important agricultural commodity have consolidated resources to produce a microarray containing 13,000 features representing approximately 12,000 different mRNAs. These are now available to academic researchers through genomics@fhcrc.org. This array overlaps previous chicken immunology arrays and extends the coverage to 24 different tissues or cell types. In conjunction with the recent release of the chicken genome sequence, this tool will have wide application to studies in developmental biology, immunology, vaccine application, as well as identification of well-characterized complex traits. The availability of genomics tools will enhance the further development of the chicken as a powerful biological model.

## Materials and methods

### Libraries and array construction

BBSRC and UD clones were shipped to the FHCRC core genomics lab. Information on the libraries joined to produce this collection is available at individual web sites and previous publications [1,6,11,15]. Microarrays were constructed using modified protocols of those discussed by De Risi *et al*. [17]. Individual PCR products were verified as unique via gel electrophoresis and purified using the Millipore Multiscreen-PCR filtration system. Purified PCR products were mechanically "spotted" in 3X SSC (1X = 150 mM sodium chloride, 15 mM sodium citrate, pH 7.0) onto poly-lysine coated microscope slides using a GeneMachines OmniGrid high-precision robotic gridder (Genomics Solutions, Ann Arbor, MI). The array layout consists of 32 blocks in a 4 × 8 configuration and each PCR product is represented once on the array. In addition, each array sub-grid (i.e., "block") contains spots representing 4 different Arabidopsis genes (negative controls) and 1 spot consisting of sheared chicken (white leghorn) genomic DNA.

A GenePix scanner-compatible file (chicken 13k_v1.0.gal) is available on line [12]. For other scanners, this file can be opened in a text editor and used to construct a similar file that meets other image analysis software's format specifications.

### RNA preparation, labeling and hybridization

Total RNA was prepared using Qiagen (Chatsworth, CA) RNeasy kits and amplified using a linear T7 promoter-based mRNA amplification method incorporating amino -allyl dUTP followed by random primer labeling with Cy™3 or Cy™ 5 (Amplification and labeling kits are available from Ambion, Inc., Austin, TX).

For hybridization, 10%, sodium dodecyl sulfate (SDS), 0.6 μl was added to the labeled RNA and heated at 99 C for 2 min. RNA was then centrifuged at 14,000 rpm for 3 min, and the sample cooled to room temperature. After placing an array slide in a hybridization chamber, 10 μl 3X SSC was added to the slide, away from the spotted area. RNA sample was then added to the array area and the cover slip promptly positioned over the array. The sealed hybridization chamber was incubated in a water bath at 63 C for 16 h. The slide was then washed for 2 min in a standard slide washing container, first in 1X SSC/0.03% SDS, then in 1X SSC, followed by a 20 min wash with agitation (60 rpm) in 0.2X SSC and a 10 min wash with agitation in 0.05X SSC. The slide was protected from light during the prolonged washes. The slide was then centrifuged (500 rpm × 5 min) to dry. Fluorescent array images were collected for both Cy3™ and Cy5™ using a GenePix 4000A fluorescent scanner (Axon Instruments, Inc., Foster City, CA) and image intensity data was extracted and analyzed using GenePix Pro 3.0 microarray analysis software.

## Authors contributions

JB and PN generated UD and FHCRC clones, respectively. DB provided the BBSRC clones. JB and JT performed the analysis for clone selection. JD and RB fabricated the microarrays. PN, JD, RB performed the analysis and validation of the microarray. MA generated the annotation file.
